# Effects of Cannabinoid Exposure during Neurodevelopment on Future Effects of Drugs of Abuse: A Preclinical Perspective

**DOI:** 10.3390/ijms22189989

**Published:** 2021-09-15

**Authors:** Aaron Mark Farrelly, Styliani Vlachou

**Affiliations:** Behavioural Neuroscience Laboratory, Neuropsychopharmacology Division, Faculty of Science and Health, School of Psychology, Dublin City University, Glasnevin, D09 W6Y4 Dublin, Ireland; aaron.farrelly@dcu.ie

**Keywords:** cannabis, neurodevelopment, perinatal, adolescence, opioid, amphetamine, tolerance, sensitization, self-administration

## Abstract

The endocannabinoid system plays a central role in the earliest stages of embryonic, postnatal and adolescent neurodevelopment. Aberrant activity of this system at key developmental phases has been shown to affect neural development. The aim of this review is to synthesise and analyse preclinical insights within rodent populations, focusing on the effects that perinatal (embryonic, gestational and early postnatal developmental stages) and adolescent (postnatal day 21–60) cannabinoid exposure impose across time on the subsequent activity of various drugs of abuse. Results in rodents show that exposure to cannabinoids during the perinatal and adolescent period can lead to multifaceted behavioural and molecular changes. In the perinatal period, significant effects of Δ^9^-THC exposure on subsequent opiate and amphetamine reward-related behaviours were observed primarily in male rodents. These effects were not extended to include cocaine or alcohol. In adolescence, various cannabinoid agonists were used experimentally. This array of cannabinoids demonstrated consistent effects on opioids across sex. In contrast, no significant effects were observed regarding the future activity of amphetamines and cocaine. However, these studies focused primarily on male rodents. In conclusion, numerous gaps and limitations are apparent in the current body of research. The sparsity of studies analysing the perinatal period must be addressed. Future research within both periods must also focus on delineating sex-specific effects, moving away from a male-centric focus. Studies should also aim to utilise more clinically relevant cannabinoid treatments.

## 1. Introduction

Cannabis is one of the most frequently used recreational drugs across the globe [1]. While recreational use is common, cannabis has also been approved as a valid treatment for various diseases, thus increasing its use amongst specific cohorts for medical reasons [2]. Past research has demonstrated that there are actually few distinct differences between medicinal and recreational cannabis users [3], but it is important to note that cannabis can be employed for purely medicinal purposes.

Cannabis itself is made up of molecules known as cannabinoids which have been utilised for millennia without any knowledge of the molecular mechanisms through which they work [4]. Currently, more is known about cannabinoids, owing to pioneering work on the cannabis plant initiated in the 1960s [5,6]. A key discovery regarding cannabis was the isolation and characterisation of delta-9-tetrahydrocannabinol (Δ^9^-THC) [6]. Δ^9^-THC is responsible for imbuing cannabis with its psychoactive proclivity [7]. Other molecules contained in cannabis, such as cannabidiol (CBD), are not psychoactive [8]. Due to the variety of molecules contained in cannabis, it has a varied effect profile. Therapeutic efficacy has been ascribed to some of its molecules (resulting in the aforementioned medicinal categorisations) [9,10] while detrimental effects such as psychosis, anxiety, depression and addiction [11,12,13,14] are also ascribed. Cannabis is a drug that has clear medicinal potential juxtaposed with equally clear negative effects. As a result of this double edge, there is a necessity on researchers to understand both the beneficial and detrimental effects of cannabis. This responsibility of researchers is constantly growing due to the legalisation and decriminalisation of cannabis, which increases its use across populations [15,16].

Acceptance of cannabis across various levels of society, from individuals to institutions, may be partly fuelled by the efficacy cannabis-based molecules demonstrate in the treatment of specific diseases [17,18]. In some instances, research appears to point to the positive effects cannabis can generate. These more positive effects may then be utilised to accommodate general acceptance of cannabis use, however, this process is undoubtedly multifaceted and driven by numerous factors. Regardless of why cannabis use is on the rise worldwide, it will still have significant effects, particularly within vulnerable populations. 

Pregnant women are one population where cannabis is frequently used to allay the symptoms of commonly experienced morning sickness [19,20]. While allaying symptoms overtly in this scenario, the aforementioned double edge of cannabis is highlighted. For example, it is known that the main psychoactive ingredient of cannabis, namely Δ^9^-THC, is of a lipophilic nature [21]. Due to this, it has been estimated that one third of the Δ^9^-THC concentration absorbed by the mother to intentionally reduce the symptoms of morning sickness inadvertently crosses the fetoplacental barrier, thus affecting the developing embryo [22]. 

Adolescence is also a period of development associated with cannabis experimentation and use for varied reasons by segments of the population [23]. During adolescence, the central nervous system (CNS) is still developing, and cannabis use at this key developmental juncture alters normative neurodevelopmental processes [24,25]. The developing adolescent brain is especially sensitive to disruption at this stage when compared to the developed adult brain [26,27]. Due to this heightened sensitivity toward cannabis use throughout adolescence as a result of intensive neurodevelopment occurring in key brain areas, adolescence can also be characterised as a key risk stage. Considering the risks imposed by cannabis use throughout pregnancy (perinatal period) and adolescence, there is a need to investigate particular responses to cannabis insult during these developmental periods [28].

The incumbent risk of cannabis use at both perinatal and adolescent stages of development stems from the fact that the system which regulates cannabinoid activity, namely the endocannabinoid system (ECS), has also been shown to regulate numerous processes central to normative neurodevelopment at these stages [29,30]. Therefore, by using cannabis for any reason, specifically at the key developmental stages previously highlighted, a person can dysregulate the finely tuned activity of this ECS, thus affecting neurodevelopment. 

Early longitudinal studies in human infants with prenatal cannabis exposure have demonstrated various aberrant manifestations such as exaggerated startle responses and poor habituation to novel stimuli [31,32]. Hyperactivity, inattention and impaired executive function have also been observed in adolescent populations [33]. The effects of early exposure to cannabis are not limited to these previously mentioned domains. It has been hypothesised that cannabis exposure at key developmental junctures can alter the effects of drugs ingested at a future time point. The significance of such an effect is very serious, the main reason being that it may leave a person more susceptible to addiction based off the altered pharmacokinetics a drug may have as a result of cannabis exposure [34]. With the weight of this situation clear, it would be expected that much research has been conducted in the area, however, experiments specifically addressing these effects remain sparse.

While a dearth of literature on the subject remains consistent, there is a general agreement that cannabis exposure during key developmental stages such as the perinatal period and adolescence can affect reactions to other drugs later in life [35]. Utilising animal models, particularly in rodents, investigations have demonstrated the effects that cannabis exposure can have on the future behaviour and molecular reactions of rodents toward opiate stimuli [36,37,38,39,40,41]. Investigations have also analysed the effects of early cannabis exposure on the subsequent behavioural reactions to, and the molecular activity of, amphetamines, cocaine and alcohol [42,43,44,45,46]. This review aims to synthesise and analyse the results of preclinical investigations that set out to determine these effects. Furthermore, the review intends to particularly delineate results based on the perinatal stage of development and the adolescent stage of development. Before a presentation of relevant literature occurs, it is salient to illustrate the role of the ECS in neurodevelopment, thus providing a mechanism for how aberrant neurodevelopment may occur as a result of cannabinoid ingestion.

### 1.1. The Endocannabinoid System and Its Functional Roles 

The ECS consists of three main components that give it the ability to modulate neural activity in the developed mammalian brain. These components are receptors, ligands and enzymes. The ECS receptors are CB_1_ and CB_2_, which are both G-protein-coupled receptors (GPCRs) [47]. CB_1_ receptors are primarily expressed in the CNS, while research demonstrates that CB_2_ is more often expressed throughout the immune system [48]. Research has also shown CB_2_ expression to occur in the CNS [49,50,51]. In tandem with receptors, the ligands of the ECS known as anandamide (AEA) and 2-arachidonyl glycerol (2-AG) are responsible for signal transduction. Many hypothesised paths are implicated in the synthesis and catabolism of AEA and 2-AG. AEA is synthesised from a phospholipid precursor known as N-arachidonyl-phosphatidyl-ethanolamine (NArPE) which is itself formed from N-arachidoylation of phosphatidylethanolamine via N-acyltransferases (NATs) [52,53]. 2-AG is synthesised from diacylglycerols (DAGs) with arachidonic acid on their 2-positions [54,55]. DAG precursors involved in the synthesis of 2-AG are produced from phospholipase-c-catalysed hydrolysis of phospatidylinositol [54] or from hydrolysis of phosphatidic acid [56]. 2-AG and AEA, after synthesis, are rendered inactive by enzymatic hydrolysis of their amide and ester bonds by the molecules fatty acid amide hydrolase (FAAH) and monoacylglycerol lipase (MAGL) [57,58]. It is important to highlight that the precursors utilised to form the endocannabinoids (eCBs) 2-AG and AEA are acted upon by specific and important enzymes. For example, 2-AG synthesis from precursor molecules is specifically catalysed by two sn-1-selective DAG lipases (DAGLs) [59]. Alternatively, AEA synthesis from the aforementioned precursors is catalysed by phospholipase D [60]. Other non-canonical pathways for eCB synthesis have been recently characterised, highlighting other potentially important molecules in ECS functioning [61,62]. Interestingly, and pertinent to this review, recent research has demonstrated how targeting specific levels of complex ECS signaling can prove beneficial. By inhibiting DAGL in rodents, researchers reduced overall alcohol consumption in a model of alcohol use disorder [63]. Additionally, it has been demonstrated that consumption of dietary fats alters the subsequent function of previously mentioned ECS units. This has various effects, including the modulation of endocannabinoid tone, resulting in neuroprotective/inflammatory effects [64]. Importantly, this recent research highlights the place of ECS signaling in the reward-related molecular activity and phenotypic behaviour of rodents as well as how other important factors (such as diet) can influence their activity. While a detailed exposition of the ECS in the developed brain was important, it is equally important to note that the ECS does not always function in the manner previously described. While its components remain relatively consistent, its function is drastically different during nervous system development.

### 1.2. The Endocannabinoid System during Perinatal Development 

The ECS has been observed operating at early stages of mammalian development. It has been observed in the preimplantation embryo and uterus [65], the placenta [66] and the developing foetal brain [67]. G-protein-coupled CB_1_ receptors have been observed as early as week 14 of gestation with receptor expression occurring across diverse brain regions [68,69]. Endogenous ECS ligands are also present at these nascent developmental stages. AEA is observable at varying concentrations from the perinatal period up to adolescence [70]. 2-AG is also present during the perinatal period with its concentration being approximately 1000-fold higher compared to AEA during brain development [71]. These eCBs and their concomitant concentrations are central to the mechanics which dictate nervous system development during the perinatal period. 

Specifically, these concentrations have a specific effect on the determination of growth cone trajectories during the process of axonal pathfinding [72,73]. They exert this modulatory effect on nervous system development through their relationship with CB_1_ receptors. The eCBs initiate CB_1_ receptor activation on various developing neurons of multiple types including GABAergic, glutamatergic and cortical excitatory neurons [74,75,76]. All of these neuron types essentially undergo nuanced development, with CB_1_ receptor activation central to inducing key developmental changes in the process. The receptors themselves are expressed in an atypical manner when compared to expression in developed brains. 

For example, CB_1_ receptors are enriched in long-range axonal tracts in the case of glutamatergic neurons, with this atypical expression ceasing after the perinatal period [29,77]. This atypical expression is a hallmark of the perinatal period and demonstrates the central role that CB_1_ receptor and eCB function play in normative development. In utero pharmacological blockade of CB_1_ receptors during the perinatal period in rodents impairs axonal pathfinding [77]. Observations such as this lend validity to the role of the ECS in neurodevelopment. 

Further demonstrating the fundamental cues imbued by the ECS are the enzymes responsible for degrading the aforementioned ligands AEA and 2-AG. These are MAGL and DAG. These enzymes are key components which degrade the ECS ligands in the developed mammalian brain [78], however, during perinatal development they are localised at the long-range axons of developing neural bundles. Their explicit activity at these temporal points modulates CB_1_ activity [79]. Through differential expression of these enzymes, the ECS can further modulate the activity of 2-AG and AEA. This modulation is observable when CB_1_ receptors, transported by Kinesin 1-mediated transport, are rendered inactive by the absence of 2-AG which is a direct result of increased MAGL [80]. When MAGL is not present, the restriction on CB_1_ receptor activity is lifted, thus allowing 2-AG to communicate with the CB_1_ receptor in a manner conducive to normative neural development [29]. 

What is clear from these observations is that native ECS functional units (receptors, ligands and enzymes) play a unique functional role during perinatal nervous system development. As a result of the functional role played by the ECS, any perturbation of the system’s functional units at this time may accommodate aberrant neural development. 

### 1.3. The Endocannabinoid System during Adolescent Development 

The ECS plays a simultaneous role in the modulation of neural activity and neurodevelopment throughout adolescence. Dynamic alterations occur in the system throughout adolescence [81], with these changes associated with overall increases in eCB signalling. While hypotheses point to the role of the ECS in neurodevelopment throughout adolescence, the exact mechanisms through which eCB signalling facilitates adolescent neurodevelopment remain unknown [82]. This may be due to the fact that adolescence is a time when the ECS is most dynamic, owing to its simultaneous modulatory capacity.

During adolescence, the ECS experiences peaks in the expression of CB_1_ receptors as well as its two primary ligands: AEA and 2-AG [82]. AEA levels increase from early adolescence to adulthood, with 2-AG levels being high at both early and late adolescence, experiencing a sharp decline of expression at the midpoint of adolescence [83]. The dynamic expression of native ECS functional units throughout adolescence represents a similar pattern to the perinatal period where similar occurrences can be observed accommodating neural development. This similarity in the mechanics of ECS expression between adolescence and perinatal periods points to the fact that the ECS may play a key role in the development of neural circuitry at adolescence. Development of this nature is essential for ensuring balanced proliferation of both inhibitory and excitatory neurons across brain regions [82]. In order to achieve this balance, adolescent neurons utilise the ECS to guide neurodevelopment.

Adolescent neurons express CB_1_ receptors to allow communication relating to neurodevelopmental trajectories to take place [84,85]. In a similar manner to the perinatal period, dynamic expression of these functional units allows for specific developmental trajectories to be adhered to. An example of this ECS modulation of neurodevelopment can be seen manifested during adolescence with significant increases in synaptic pruning. This process is known to increase the efficiency and selectivity of information processing [86,87]. This pruning occurs in an imbalanced manner with more excitatory synapses being pruned, resulting in more inhibitory neurotransmission [88]. The ECS is central to regulating this pruning process and through continuous modulation, on varying neural types, the ECS ultimately aids the developing nervous system in strengthening and eliminating synaptic connections as necessary [89,90]. These maturational processes such as synaptic pruning are hallmarks of adolescence, and the ECS appears to be a key system which aids in the process by providing necessary signals to specific neurons. The role of the ECS in the process of synaptic pruning can be further observed via administration of CB_1_ receptor antagonists. Antagonising this key functional unit of the ECS in adolescent rodents was observed to significantly prevent natural decreases in postsynaptic density from occuring, thus suggesting CB_1_ receptors’ responsibility in the accommodation of pruning and overall neurodevelopmental processes [81,91]. 

While these aforementioned observations point to a potential role for the ECS in adolescent neurodevelopment, little is known about the exact mechanisms the ECS utilises to bring about these neuronal alterations [91]. It is clear, however, that the system plays a dynamic role in the adolescent brain, particularly as it relates to mesocorticolimbic structures and their development. These structures include the prefrontal cortex, nucleus accumbens (NAcc) and caudate putamen which are areas primarily responsible for expressing reward, motivation and cognitive behaviours [91]. The aforementioned processes are particularly salient to this review as these are the regions responsible for mediating the rewarding stimuli of various drugs. If ECS perturbation at key developmental stages directly affects neurodevelopment as discussed, these effects may alter how reward structures such as the NAcc subsequently react to various drugs in the future. Such alterations will be discussed in the next section. 

### 1.4. ECS Perturbation Results in Neuronal Alterations

Developmental exposure to cannabinoids in rodents at perinatal stages of development is associated with changes in numerous neurotransmitter systems [43,92,93]. These changes in development are a direct result of exogenous cannabinoids dysregulating the previously illustrated neurodevelopmental trajectories which the ECS modulates. It appears that perinatal cannabinoid exposure affects the subsequent development of monoaminergic, dopaminergic and GABAergic systems. Research on each of these systems demonstrates this. Analyses focusing on monoaminergic nerve types suggested that Δ^9^-THC positively affects future concentrations of brain amines [92,93,94]. Perinatal perturbation of the ECS also altered subsequent neurodevelopment in such a manner that presynaptic dopamine receptor responses were enhanced, whilst postsynaptic dopamine receptor responses were reduced [35]. Such alterations in receptor functionality are not limited to dopaminergic neurons either. Clear transformations have also occurred in serotonergic [93,95], glutamatergic [96,97,98] and GABAergic neurons [99,100]. These transformations ultimately affect the future capability of these tracts to process neural information across diverse brain regions. 

It is clear from these results that Δ^9^-THC may be playing a role in the perturbation of normative neuron development. These variations in the development of various neuronal systems subsequently generate compensatory alterations at the receptor level. Within the scope of this review and its focus on the activity of other drugs, direct changes at the receptor level are particularly salient as they may alter how other drugs function at future time points. Receptor alterations such as these have been observed recently by researchers [38], with such changes observable in both the pre and postsynaptic areas [35]. 

Adolescent exposure to cannabinoids also has untoward effects in terms of the development and future activity of specific neuron types. Research has demonstrated fewer dopaminergic neurons, increased dopamine turnover and a concomitant reduction in dopaminergic activity as a result of adolescent Δ^9^-THC exposure [101,102]. Rodents treated with the CB_1_ agonist WIN55,212-2, for example, demonstrated increased dopaminergic turnover, particularly in the dorsal and ventral striatum [103]. Similar to the perinatal stage, other neuron types are also affected such as glutamatergic and GABAergic neurons. The normative function of these neuron types in adulthood is significantly disrupted by Δ^9^-THC exposure during adolescence [104]. In the PFC, the glutamatergic system was also shown to be affected by adolescent Δ^9^-THC exposure. Increases in native glutamatergic functional units, as well as diminished levels of mGlu5, were observed in adult rodents exposed to Δ^9^-THC in adolescence [81,105]. GABAergic nerve types were also observed to have a reduction in communication as a result of Δ^9^-THC exposure in adolescence [106]. These alterations of integral neural systems at a molecular level may also manifest phenotypically, resulting in changes to behavioural patterns surrounding drug use. These changes at both molecular and behavioural levels will become clearer when specific research in the area is presented. 

## 2. Studies Examining the Impact of Adolescent and Perinatal Cannabinoid Exposure on Future Drug Use Effects

The following studies utilise various animal models and particular strains of rodents in order to draw conclusions about the effects of cannabinoid exposure on the subsequent activity of other drugs. 

Specifically, studies within this review utilised rodent strains (primarily rats). Common strains, such as Sprague-Dawley and Wistar, are frequently used. Some studies also utilise less common strains, such as Fischer 344, Lewis and Long Evans rats. While all of the aforementioned strains fall under the genus *Rattus Norvegicus*, it is important to consider the slight reactive variations that may occur across strains. It has been demonstrated previously that specific strains may be optimal for specific experimental designs [107]. Therefore, upon interpretation of this review it should be noted that results have not been obtained across a consistent rat strain and results within a particular strain are not fully generalisable to other strains.

One study contained in this review also utilises Institute of Cancer Research (ICR) mice. These results with ICR mice were deemed pertinent to the specific aims of this review as they reflected the effect of cannabinoids on the future activity of drugs. Therefore, the study was included in the analyses, but it must be highlighted that ICR mice are a different species, and belong to a genus separate from rats, known as *Mus Musculus.* It is known that this genus reacts in a unique manner to various stimuli when compared to *Rattus Norvegicus* [108]. These species specific reactions are also worth considering when interpreting studies contained within this review. 

Animal models of drug intake are crucial and informative tools for the exploration and subsequent identification of pathological molecular mechanisms and drug targets [109] (please see Table 1). Recently, rats have been hypothesised as optimal models in drug research with high translation of results obtained in this species to humans—which is ultimately the main goal of animal research [108]. While rats have been identified as optimal in some instances, data obtained in mice can still provide salient information and guide early progress across various outcomes. Therefore, rats and mice have been included in this review. While it is never expected that an animal model can provide a direct representation of the human condition, this review’s aims can be achieved via inclusion of both rats and mice. By including both species of rodents, a more holistic picture of the effects cannabinoids have on subsequent drug activity can be obtained. 

In tandem with the animals used, the various models built to measure specific behaviours and activities are important. Numerous models have been created and validated to this end, with the two most commonly utilised within studies included in this review being the self-administration (SA) and conditioned place preference (CPP) paradigms. The elevated plus maze (EPM) paradigm and intracranial self-stimulation (ICSS) paradigm are also used but less often in studies contained within this review. A brief summary of each paradigm is important. 

SA paradigms allow for the objective study of drug-related behaviours [113]. SA paradigms can be characterised by non-operant and operant methods [114]. Non-operant procedures are usually (but not always) limited to oral administration of a drug (commonly used in studies on alcohol), while operant-based methods rely on reinforcement via specific administration schedules [114]. In an SA experiment, an animal is confined to a specifically built ‘chamber’ or ‘box’ where they must perform particular acts (pressing a button/lever) in order for a drug to be delivered to them. Studies contained in this review most commonly utilise an intravenous catheter for drug delivery, thus when it is stated that animals were prepared for SA, it is referring to the catheter preparation. Overall, SA paradigms are useful because they have a high correspondence with drug related behaviours. This correspondence gives them good face and construct validity, thus allowing for the meaningful translation of results from animal models to humans [113]. For a comprehensive description of particular SA methods, consult the aforementioned references [113,114].

Another paradigm which is commonly utilised within experiments of this review is the CPP paradigm. Similar to SA methods, CPP is carried out within a specifically designed chamber, being either a two or three-compartment conditioning box [114]. In this method, the motivational properties of drugs can be analysed via pairing specific treatments with a previously neutral set of environmental cues [115]. Essentially, this paradigm results in a specific environment (chamber) being paired with drug administration and vehicle administration separately. After such pairing occurs, animals are placed back in the environment in a drug-free state and tested for their preference for each environment [110]. The time an animal spends in a particular chamber will be used as the dependent variable, ultimately reflecting a conditioned preference. Overall, the proclivity an animal demonstrates towards either the drug-paired chamber or the vehicle-paired chamber in a drug-free state becomes the basis for researchers’ conclusions on the rewarding effects of particular drugs [115,116]. For a comprehensive analysis of CPP, consult the aforementioned citations [114,116]. 

The EPM is another paradigm used less often in studies contained within this review. It is a versatile paradigm capable of obtaining information pertaining to the behavioural and molecular underpinnings of anxiety. It has good face and construct validity, making it a common choice amongst researchers [111]. In practice, a rodent is placed in the centre of a four-armed elevated maze apparatus and observed. There are two open arms and two closed arms in the device. Time spent within the open arms and the number of single entries to the open arms are used as the dependent variable in this paradigm. This variable is based on anxiety/avoidant behaviour, and when coupled with molecular analyses, this paradigm becomes a powerful tool for understanding this specific behaviour further. 

ICSS is utilised by one study included in this review. This is an operant behavioural paradigm where animals deliver specific electrical impulses to varying reward pathways in their own brain. These reward pathways are hypothesised to mediate both natural and ICSS rewards [112]. A significant strength of using ICSS methods is that the paradigm can provide researchers with more accurate representations of reward processes due to the control given to researchers by the paradigm. ICSS reward can also be measured with much more specificity than natural reward [112]. However, this artificial reward essentially bypasses various inputs which are central to a more holistic view of reward processing [112], thus it is not without limitations.

The previously described models are used in studies contained within this review to determine the effects of developmental cannabinoid exposure on future drug activity. With the animal strains and paradigms sufficiently explained, experiments where they are utilised will now be presented. 

### 2.1. Perinatal Exposure to Cannabinoids

A brief account of studies focusing on the effects of perinatal cannabinoid exposure on subsequent drug use is presented in Table 2. For the purposes of this review, the perinatal period will include the embryonic period, gestational period (for information pertaining to gestational period length with concomitant equivalents consult Table 3) and early postnatal days [117]. Such a range allows for the meaningful capturing of various salient experiments that analyse the effects of cannabinoid exposure on the subsequent behavioural and molecular effects of specific drugs of abuse. Included studies have been particularly focused on opiates and amphetamines, with one study utilising cocaine. The presentation of analyses will begin with opioids, specifically morphine. 

In this experiment [94], pregnant Wistar rats were exposed at daily intervals to Δ^9^-THC from day 5 of gestation (see Table 3 for gestation information) to postnatal day 24 when the pups were weaned. Rats were subsequently subjected to a dose of 5 mg/kg Δ^9^-THC. The control group was subjected to a sesame oil vehicle. After Wistar rats were subjected to Δ^9^-THC for the majority of gestation, they were left to mature for 7–10 weeks before being segregated into different experimental groups based on gender and treatment type. One group of these mature pups were subjected to a morphine SA task, whilst another group were subjected to morphine SA, receiving a saline dose on the final day of testing to eliminate the morphine response. Across SA paradigms, it was observed that females reached higher and more consistent patterns of morphine administration when compared to male pups. Investigators suggest that these higher patterns observed in female pups correspond with higher dopaminergic activity in the NAcc prior to the onset of morphine SA as compared to male pups. Investigators also observed lower dopaminergic activity in Δ^9^-THC-exposed females when compared to vehicle-treated females, thus demonstrating a desensitising effect of Δ^9^-THC on dopaminergic activity with subsequent effects on morphine SA. Overall, it was concluded that female and male Wistar pups perinatally exposed to 5 mg/kg Δ^9^-THC or vehicle performed equally in a morphine SA paradigm. Δ^9^-THC did not significantly affect the reinforcing efficacy of morphine in males or females.

More studies further aimed to delineate the effect of Δ^9^-THC on subsequent opioid-related behaviour. Focusing specifically on heroin, investigators exposed pregnant Long–Evans rats to 0.15 mg/kg Δ^9^-THC from gestational day 5 to postnatal day 2 [39]. Another group was subjected to vehicle treatment during this time. Upon birth, pups from both treatment groups were cross-fostered. On postnatal day 21, male offspring were weaned and housed until postnatal day 55 where they were prepared for SA paradigms. All pups were allowed to self-administer a heroin dose of 15 µg/kg under a fixed ratio. After 6 days, this dose was changed to 30 µg/kg. The dose was altered depending on group membership. Δ^9^-THC- and vehicle-exposed pups from postnatal day 2 and postnatal day 62 were then sacrificed with their brains stored for analyses. These analyses revealed similar heroin intake for both groups of Long–Evans pups, with Δ^9^-THC-exposed pups exhibiting shorter latency to first active lever press. The Δ^9^-THC group also responded more positively to lower heroin doses and exhibited higher heroin-seeking behaviour. Overall, this investigation revealed effects of perinatal Δ^9^-THC exposure which alters subsequent heroin-seeking behaviour in male Long–Evans pups. The authors allude to various disruptions at a molecular level which can aid in explaining the previously observed behaviours. It seems Δ^9^-THC exposure significantly affects proenkephalin messenger RNA (mRNA) expression during the prenatal period, with these alterations directly affecting the opioid system’s functioning.

The effects of Δ^9^-THC exposure on subsequent opioid activity have been further analysed [38]. Specifically, potential effects on the regulation of NAcc dopamine receptor mRNA were analysed in Long–Evans rat offspring. Rodents determined to be pregnant were housed alone and either treated with 0.15 mg/kg Δ^9^-THC or vehicle from gestation day 5 to postnatal day 21. On postnatal day 2, Long–Evans pups were raised by other maternal figures. On postnatal day 21, the pups were weaned and allowed to undergo maturation. Only male Long–Evans pups were brought forward for analysis. At postnatal day 62, male rats were sacrificed with their brains subsequently harvested and stored for analysis. Researchers observed significant molecular alterations as a result of Δ^9^-THC exposure. Observations pointed to significant chromatin immunoprecipitation of the offspring brains, specifically revealing an increase in 2meH3k9 repressive mark with concomitant decreases in 3meH3K4 and ribonucleic acid (RNA) polymerase II at the dopamine receptor 2 gene locus. This was all observed in Δ^9^-THC-exposed male offspring. It is worth noting that decreased dopamine receptor 2 expression was accompanied by a reduction in dopamine receptor 2 binding sites, with accompanying increases in opiate sensitivity lasting into adulthood. Molecular observations of this nature have been replicated recently in female adolescent populations exposed to Δ^9^-THC [119].

Investigations have also extended analyses within the realm of opioids across generations [118]. In this experiment, twenty one-day-old Long–Evans males and females were obtained and housed in same sex groups of four. These animals were subsequently administered with 1.5 mg/kg Δ^9^-THC via injection every third day between postnatal day 28 and 49. Δ^9^-THC-exposed females were then mated with Δ^9^-THC-exposed males. Long–Evans rats were also vehicle treated and mated accordingly. Females were then singly housed during pregnancy with mixed litters established across Δ^9^-THC and vehicle variables. Drug-naïve mothers were then utilised as nursing mothers, with the new litters weaned at postnatal day 24. Heroin SA was then conducted in new generation groups. Groups had access for three hours a day to 30 µg/kg of heroin via intravenous administration. Results showed no differences related to parental Δ^9^-THC exposure on heroin SA behaviour. However, when the work effort necessary to obtain heroin was increased, the offspring of parents who experienced Δ^9^-THC perturbation perinatally exhibited significantly increased work effort to obtain heroin. Through open field analysis, investigators confirmed that the progeny of Long Evans rats exposed to Δ^9^-THC manifested with behavioural disturbances, making it more likely for these offspring to seek out heroin. The authors suggested that exposure to Δ^9^-THC alters molecular regulation in the striatum, thus altering the organism’s sensitivity to compulsive drug intake in Δ^9^-THC-naïve offspring.

Amphetamines are another class of drug to which analyses have been extended. An investigation specifically explored these effects in Sprague Dawley rats [43]. In this investigation, female Sprague Dawley rats were administered with 0.15 mg/kg Δ^9^-THC or vehicle. This administration began at gestation day 1, lasting until gestation day 21. Subsequent litters were then culled to 10 pups. Pups were then weaned on postnatal day 21 and subsequently subjected to an array of challenges, with the amphetamine challenge being most pertinent to this review. At postnatal day 60, one male and one female were given an amphetamine challenge. Activity monitors were used to assess locomotor activity before and after amphetamine administration. Postnatal day 60 male and female pups were pre-recorded for 15 min to establish baseline activity levels. They were then administered with 1 mg/kg amphetamine sulphate and recorded for 60 min. This test was repeated to include a total of 35 animals with the vehicle pre-treated group containing 11 males and eight females. The Δ^9^-THC group contained nine males and seven females. Baseline measurements demonstrated no significant litter effect, however, there was a clear sex effect with female rats observed with significantly greater scores on the dependent variable (distance travelled) compared to males. Importantly, there was no significant effect of Δ^9^-THC on baseline activity. After amphetamine was administered, there was again a significant sex effect with females travelling further than males once again. Interestingly, treatment with Δ^9^-THC also significantly affected scores on the dependent variable. It was observed that Δ^9^-THC-exposed pups had a dampened locomotor response to amphetamines across sexes. No interactions between sex and Δ^9^-THC treatment were observed, suggesting that initial treatment with Δ^9^-THC may have reduced overall locomotor activity across sexes. Researchers suggested that the activation of CB_1_ receptors in this instance dampened the increase in dopaminergic output, thus altering results on the dependent variable.

Another study focused specifically on cocaine but Δ^9^-THC was not utilised, but rather the FAAH inhibitor URB597 was used to determine whether perinatal exposure to URB597 alters the subsequent activity of cocaine [44]. ICR mice were used in this instance, with pregnant mice producing offspring. These pregnant mice were assigned to either a URB597 treatment group or a vehicle group. Groups were then administered with 1, 3 or 10 mg/kg URB597 depending on their grouping. Pregnant mice being used for the cocaine challenge were injected daily from embryonic day 10.5 (measured from conception) to postnatal day 7. On postnatal day 21, pups were weaned and housed in groups of three to five. The ICR pups were then tested using the CPP procedure. Only male ICR pups were used in the CPP. Eight conditioning trials were conducted on separate days with ICR pups being divided into two equal groups: treated with vehicle and URB597. Mice in each group were administered with either saline solution (vehicle) or 10 mg/kg cocaine. Both cocaine and vehicle were administered intraperitoneally. The percentage of time that mice spent in the drug-paired area of the CPP box represented the dependent variable in this investigation. Analysis of data revealed no significant effect of URB597 on cocaine-related activities. However, post hoc analysis revealed a significant CPP for vehicle-treated mice, but not URB597-treated mice. Post hoc analyses essentially revealed that mice perinatally exposed to vehicle spent significantly more time in drug-associated environments. This preference was not observed in URB597-treated mice, demonstrating that this ECS modulator may have decreased the rewarding effects of cocaine. Researchers concluded that, overall, URB597 produced less serious behavioural consequences compared to Δ^9^-THC.

The effects of perinatal exposure to cannabinoids and its effects on alcohol-related behaviours have also been investigated. One investigation in this area utilised Wistar rats, involving multiple experiments to understand the relationships between perinatal Δ^9^-THC exposure and ethanol SA [42]. In one experiment, pregnant Wistar rats were treated daily with Δ^9^-THC (5.0 mg/kg) and alcohol (3%), Δ^9^-THC (5.0 mg/kg) and sucrose (4.2%), vehicle and alcohol (3%) or vehicle and sucrose (4.2%). Pregnant dams were treated with these combinations from gestational day 15 to postnatal day 9. Subgroups of ethanol-exposed rats were utilised to measure blood alcohol levels on day 20 of gestation and postnatal day 9. After treatment and measurement, litters were reduced to a standard size of six male pups which would be utilised in subsequent experiments. Preliminary results in this instance demonstrated that Δ^9^-THC treatment did not significantly modify ethanol consumption in pregnant dams during the gestational or postnatal period. 

The same investigators carried out another experiment to evaluate whether exposure to Δ^9^-THC, ethanol or a combination of the two during the perinatal period influences the reinforcing properties of ethanol in adulthood [42]. Further, the researchers wanted to determine the effects of the CB_1_ receptor antagonist SR-141716A on ethanol SA. Two-month-old Wistar rats, which had been perinatally exposed to Δ^9^-THC, ethanol or a combination of the two, were trained to self-administer 10% ethanol 30 min daily under a fixed-ratio 1 (FR1) schedule of reinforcement [121]. This schedule was adhered to for 23 days and upon completion, the effect of Δ^9^-THC (and SR-141716A) on ethanol self-administration was evaluated. The original group was split into four, with one group intraperitoneally injected 30 min prior to the SA session with SR-141716A at doses of 0.3, 1.0 and 3.0 mg/kg (or vehicle in same dose). Analyses did not reveal any significant differences in ethanol SA between different groups of Wistar pups. Interestingly, administration of the CB_1_ receptor antagonist SR-141716A dose-dependently reduced response to ethanol SA, with analyses revealing an overall significant effect of the CB_1_ receptor antagonist. Doses of 1.0 and 3.0 mg/kg significantly reduced the Wistar pups’ response to ethanol SA. Conclusions pointed to the fact that rodent pups received pharmacologically relevant doses of ethanol, Δ^9^-THC or a combination of the two, whilst displaying no overt signs of effect on subsequent ethanol SA behaviours. Results regarding SR-141716A align with previous observations that treatment with a CB_1_ antagonist can prevent the reinstatement of alcohol seeking in rats. 

### 2.2. Adolescent Exposure to Cannabinoids

A brief account of studies focusing on the effects of adolescent cannabinoid exposure on subsequent drug use is presented in Table 4. Within rodent populations, adolescence is typically defined as a range between postnatal day 21 and 60, which can be further broken down into early adolescence (postnatal day 21–34), middle adolescence (postnatal day 34–46) and late adolescence (postnatal day 46–59) [122]. All studies representing adolescence in this review contain rodents within the aforementioned age range. Exposure to cannabinoids within these sensitive age ranges has been hypothesised to alter the behaviours toward, and subsequent molecular activity of, opioids and amphetamines [123]. 

Via specific analysis of dopaminergic neurons, investigators specifically sought to determine if exposure to cannabinoids during adolescence affected dopaminergic responses to other drugs of abuse in the ventral tegmental area (VTA) of rodents [123]. In this study, male Sprague Dawley rats were housed in two separate groups of different ages, an adolescent group (aged 5–6 weeks) and an adult group (aged 8–9 weeks). Rodents in both of these groups received intraperitoneal injections twice daily for three days. The dose of the cannabinoid agonist WIN55,212-2 in the injection was escalated over the three-day period (Day 1: 2.0 mg/kg, Day 2: 4.0 mg/kg, Day 3: 8.0 mg/kg). In the same schedule, the control group rats were treated with vehicle. Fourteen days after the last WIN/vehicle administration, in vivo extra cellular single-unit electrophysiologic recordings were performed in rodents prepped for such analyses. Baseline activity across various neurons was initially determined with a total of 107 dopaminergic neurons included in the analyses primarily located in the VTA and NAcc. After a drug-free period, rodents were subjected to various challenges. Data revealed that cannabinoid-exposed rodents showed a reduced response to further WIN55,212-2 challenges. Further, the adolescent cannabinoid-exposed group displayed long-lasting tolerance to opioids and amphetamines. This was established when morphine administration (0.5–8.0 mg/kg) and amphetamine administration (0.0625–2.0 mg/kg) caused minimal effect on VTA dopaminergic activity 2 weeks after WIN55,212-2 exposure. 

Studies focusing on opioids have further replicated the effects of Δ^9^-THC in adolescence [40]. Male Long–Evans rats were exposed to Δ^9^-THC (1.5 mg/kg) or vehicle from postnatal day 28–49. Δ^9^-THC was administered every third day to mimic the intermittent use characteristic of adolescence. After Δ^9^-THC exposure was completed, rodents were subjected to SA paradigms. Analyses revealed that Δ^9^-THC-exposed rats presented with an upward shift in heroin SA, whereas control animals maintained a consistently stable pattern once peak heroin intake (30 µg/kg) was established. It was observed that Δ^9^-THC-treated animals continued to shift their heroin intake upward, achieving 25 total responses compared to 15 total responses in the control group. This heightened proclivity towards heroin was also observed in the Δ^9^-THC-exposed group via their heightened opiate consumption during the maintenance phase of the SA paradigm. These observations were corroborated by investigators’ post mortem brain analyses. Specifically, researchers observed increased striatal preproenkephalin mRNA expression in the NAcc of Δ^9^-THC-exposed Long–Evans adolescents. Opioid receptor activity was also observed to be potentiated in key reward pathways of Δ^9^-THC-treated rats. Taken together, these conclusions point to similar molecular perturbations observed in perinatal research.

Further data pertaining to Δ^9^-THC were obtained when adolescent Wistar rats were divided into two groups, receiving escalating doses of Δ^9^-THC or vehicle treatment for 11 consecutive days [41]. Treatment in this manner began on postnatal day 35 and lasted until postnatal day 45. Administrations occurred twice daily on an escalating dose schedule (Day 35–37: 2.5 mg/kg, Day 38–41: 5 mg/kg, Day 42–45: 10 mg/kg). Rats were also vehicle treated to act as a control. After dosing with Δ^9^-THC or vehicle, rodents were subjected to EPM and SA paradigms. Researchers also conducted a reinstatement of heroin-seeking behaviour test using the α2 adrenoreceptor antagonist yohimbine. In the EPM, Δ^9^-THC treatment significantly reduced the percentage of time spent in open arms. In the SA paradigm, no significant effect of Δ^9^-THC was observed. Both Δ^9^-THC- and vehicle-treated animals exhibited no differences in either the acquisition of operant responding or heroin intake at baseline. Upon analysis of the reinstatement behaviour, drug seeking was shown to increase in both groups and further post hoc analyses revealed that Δ^9^-THC exposure significantly increased the heroin seeking initiated by yohimbine. Overall, this study counters previous results, observing no significant effects of Δ^9^-THC exposure on SA behaviour. Interestingly, Δ^9^-THC was shown to affect heroin seeking when acting in tandem with yohimbine. Manifesting with an increased response to the α-2 adrenoreceptor antagonist due to adolescent Δ^9^-THC exposure is an interesting result meriting further investigation.

Further work into the effect of Δ^9^-THC exposure on opiate-related behaviours and molecular activity was completed across breed in Fischer 344 and Lewis inbred rats aged between postnatal day 30 and 35 [124]. After 1 week of habituation, both breeds were divided into two experimental groups. Group members received twice daily administrations of Δ^9^-THC for 3 days, with doses escalating each day (Day 1: 2 mg/kg, Day 2: 4 mg/kg, Day 3: 8 mg/kg). Other groups received vehicle treatment in the same schedule. In vivo microdialysis was subsequently utilised to determine the responsiveness of neurons in the NAcc shell and core dopaminergic neurons. Adolescent Δ^9^-THC exposure was observed to potentiate the dopaminergic effects of heroin in the NAcc shell and core of Lewis rats. Exposure to Δ^9^-THC in Fischer 344 rats only potentiated dopaminergic activity in the NAcc core. In the CPP paradigm, control Lewis rats developed more pronounced CPP to heroin when compared to Fischer 344 rats. In the Δ^9^-THC-exposed Lewis rats, exposure did not significantly affect heroin CPP, but it did potentiate the heroin priming effect. Within the Fischer 344 strain, Δ^9^-THC exposure increased heroin CPP and made it extinction resistant. Interestingly, Lewis rats were observed to demonstrate seeking behaviours during the extinction phase, while Fischer 344 rats did not demonstrate significant seeking behaviours. 

Replication of opioid-related observations was achieved [125] when Wistar rats were mated and subsequently utilised for experimentation. Wistar pups were weaned on postnatal day 22 and subjected to a cannabinoid/vehicle administration schedule. The CB_1_ agonist CP-55,940 (CP) (0.2 mg/kg or 0.4 mg/kg) or vehicle was administered to pups once daily from postnatal day 35–45 [125]. After this administration period, pups were subjected to SA paradigms based on treatment and sex. Data revealed that adolescent cannabinoid exposure altered opioid-related activities in a sex-dependent manner. CP-55,940-treated males had a higher SA under an FR1 schedule. Conversely, in females, CP exposure during adolescence did not modify opioid-related activities under any SA schedule. Post mortem brain analyses corroborated behavioural findings from a molecular perspective. CP-55,940 exposure was observed to increase opioid receptor levels in the subcallosal streak while simultaneously decreasing receptor functionality in the NAcc. These alterations were sex specific, only observable at a significant level in males. Conclusions point to the role sex may play in mediating adolescent exposure to CP-55,940.

Interestingly, the transgenerational effects of adolescent cannabinoid exposure have been analysed in Sprague Dawley rats [126]. Researchers took female adolescent rats at postnatal day 30 and exposed them twice daily to the cannabinoid agonist WIN55,212-2 intraperitoneally at escalating doses (Day 1: 1.0 mg/kg, Day 2: 2.0 mg/kg, Day 3: 4.0 mg/kg). A group of rodents were also treated with vehicle at the same doses. At 60 days old, all females were housed and mated with colony males. All male offspring subsequently born were weaned and housed on postnatal day 21. These pups were then subjected to the CPP paradigm at 40 days old. Selectively, groups were treated with morphine sulphate (1 or 5 mg/kg) or vehicle (0.9% NaCl, 1 mL/kg) with only one animal per litter used to avoid litter effects. Upon analysis of the CPP data, it was found that adolescent offspring exposed to WIN55,212-2 in a transgenerational manner exhibited greater sensitivity to morphine CPP when compared to their vehicle-treated counterparts. A significant interaction between morphine dose and maternal drug history was revealed with doses of 1 and 5 mg/kg affecting CPP results.

These transgenerational effects of cannabinoid exposure on opiate-related behaviours have also been observed in female offspring [127]. Sprague Dawley females were treated twice daily via subcutaneous injection with escalating doses of the cannabinoid agonist WIN55,212-2. Another group of female rats also received vehicle treatment in the same dose schedule (Day 1: 1 mg/kg, Day 2: 2 mg/kg, Day 3: 4 mg/kg). Dosing occurred on postnatal day 30, 31 and 32. At postnatal day 60, these animals were introduced to a male colony to allow for mating. Subsequent litters were born and experimental groups were created (five males and five females). Female offspring were the specific focus in this instance. WIN55,212-2- and vehicle-exposed offspring were weaned on postnatal day 21 and group housed until postnatal day 60. On this day, female offspring were tested for morphine-induced locomotor sensitisation. WIN55,212-2- and vehicle-exposed offspring received either saline (0.9% NaCl, 1 mL/kg) or 7.5 mg/kg morphine sulphate. Locomotor activity was subsequently analysed. Data revealed further significant effects of developmental cannabinoid perturbation on opiate-related activities, with this perturbation transcending next generation insult. 

A number of studies have also explored whether WIN55,212-2 exposure during adolescence has effects on future amphetamine responses [128]. In one of these studies, Sprague Dawley rats were used. Rats in these experiments received cannabinoid exposure in early adolescence (Day 28–32) with concomitant amphetamine insult occurring later in adolescence (Day 40). In one experiment, 28-day-old Sprague Dawley rats were treated with WIN55,212-2 (1.25 mg/kg) or vehicle once daily for 5 days. After this administration period, a 7-day drug-free period was initiated. An injection challenge of either amphetamine (0.5 mg/kg), WIN55,212-2 (1.25 mg/kg) or vehicle was then administered interperitoneally. Dopaminergic activity in the NAcc was then analysed using *in vivo* microdialysis. The same researchers also carried out another experiment with differing dosages (0, 0.625, 1.25 and 2.5 mg/kg), again utilising WIN55,212-2, [128]. Locomotor activity and stereotypical behaviour were subsequently monitored. Results demonstrated no significant alterations of dopamine levels in the NAcc as a result of adolescent WIN55,212-2 exposure. Behavioural results demonstrate that rats exposed to WIN55,212-2 manifest with a tendency towards reduced locomotor activity when challenged with amphetamine. However, this observation was not deemed statistically significant. Similarly, WIN55,212-2 exposure at adolescence did not significantly affect stereotypical responses to amphetamine, with no rats obtaining a score above 7 on the stereotypic behaviour scale. 

Results achieving no statistical significance have been replicated by recent research analysing WIN55,212-2 exposure during adolescence [129]. Sprague Dawley rats were divided into two groups. These rats were then exposed to WIN55,212-2 or vehicle from postnatal day 42–52. An escalating dose of WIN55,212-2 was used in this study (Day 42–44: 2 mg/kg, Day 45–48: 4 mg/kg, Day 49–52: 8 mg/kg). On postnatal day 60, rats were then then assessed for locomotor sensitisation after acute cocaine administration. Rats were exposed to 10 mg/kg cocaine intraperitoneally and observed in order to determine baseline locomotor responses. Locomotor activity was indexed by distance travelled. As the rats moved into adulthood, they were subsequently subjected to, and trained in, SA paradigms via an FR1 schedule of reinforcement. After 14 sessions of SA, rats were then subjected to 14 6-h SA sessions to allow for potential dose escalations to occur. On postnatal day 130, rats were again subjected to acute cocaine administration with subsequent analysis of locomotor activity. Observations made across this experiment demonstrated cross-sensitisation to the locomotor effects of cocaine in adolescence. This cross-sensitisation did not persist into adulthood as evidenced by testing conducted on postnatal day 130 when adult Sprague Dawleys exposed to WIN55,212-2 in adolescence demonstrated similar locomotor sensitisation to vehicle-treated rats. Overall, results here further point to the fact that WIN55,212-2 is unlikely to be causally linked to future cocaine use patterns. It is worth highlighting that WIN55,212-2 exposure did significantly affect adolescent reactions to subsequent cocaine exposure. This study is limited by the fact it solely focuses on males.

Studies testing the effects of Δ^9^-THC exposure on subsequent amphetamine responses have also been conducted [128]. Twenty-eight-day-old Sprague Dawley rats were treated with Δ^9^-THC (0, 0.075, 1.5 and 3.0 mg/kg) intraperitoneally once daily for 5 days. After this administration period was completed, a 7-day drug-free period was initiated, with rats receiving an amphetamine challenge (0.5 mg/kg) after this period. Locomotor activity and stereotypical behaviour were subsequently analysed. The same researchers conducted another experiment in the same manner, only this time animals received a different dose upon amphetamine challenge (2.0 mg/kg) [128]. Locomotor activity was subsequently monitored. Data revealed that Δ^9^-THC exposure had no significant effect on subsequent responses to amphetamine challenges as measured via locomotor activity and stereotypical behaviour scales. 

A recent investigation counters these observations, showing that Δ^9^-THC exposure in adolescence potentially affects the subsequent activity of amphetamines and Δ^9^-THC itself. Sprague Dawley females were administered with Δ^9^-THC (0.1 or 1.0 mg/kg) or vehicle intraperitoneally between postnatal day 28 and 50 [130]. These Δ^9^-THC-exposed females were then mated with drug-naïve males. The offspring were then exposed to varying doses of Δ^9^-THC (0, 0.1, 0.5 and 1.0 mg/kg) and amphetamines (0, 0.1, 0.5 and 1.0 mg/kg). Through the use of ICSS, researchers were able to determine the reward-modifying effects of the previous Δ^9^-THC and amphetamine dosages in male offspring. Results demonstrated that the reward-facilitating effects of administered Δ^9^-THC at the lower dose (0.1 mg/kg) were abolished in the male offspring exposed to Δ^9^-THC across generations. The reward-facilitating effect was not altered at the higher doses (0.5 and 1.0 mg/kg). The reward-facilitating effects of all amphetamine doses (0.1, 0.5 and 1.0 mg/kg) were significantly decreased in male offspring exposed to Δ^9^-THC. It is important to highlight that these effects were observed in the absence of in utero cannabinoid exposure and conclusions point to the necessity of viewing such transgenerational alterations in the reward-related activity of Δ^9^-THC and amphetamines as potentially increasing susceptibility to addiction. 

Observations demonstrating no effects of adolescent cannabinoid exposure on subsequent amphetamine and cocaine responses have been challenged. Wistar rat litters were weaned on postnatal day 22 to form experimental groups for such experimentation [131]. These weaned pups were then administered with either CP55,940 (0.4 mg/kg) or vehicle. From postnatal day 28–38, pups received 11 injections. Rats were kept individually housed until postnatal day 75. Various experiments were subsequently carried out on Wistar pups. On postnatal day 90, both CP55,940 and vehicle-exposed pups across sexes were subjected to an SA paradigm. The SA experiment consisted of three phases: AutoShaping, FR1 (with food restriction) and FR1 (with *ad libitum* food). Initial analyses revealed no differences in bodyweight between CP55,940-treated or vehicle-treated rats subjected to the first food-reinforced task. Sex effects were observed with males weighing more than females.

A second set of both CP55,940-treated males and females, as well as vehicle-treated males and females, were subjected to the previous phases of the food-reinforced task. After being surgically prepared, this experimental group began the drug-reinforced behaviour aspect of the study on postnatal day 100. Consisting of two phases, the groups were subjected to cocaine doses (1 mg/kg) upon lever presses. Data from this second cohort revealed a significant effect of CP55,940 treatment in females, but not males. Wistar females in the CP55,940-treated group were observed to self-administer a significantly greater amount of cocaine than their vehicle-treated counterparts. Interestingly, in these experiments, a parallel food-reinforced behaviour task was run in order to test the specificity of effects observed in the SA paradigm. With food as a reinforcer, no treatment interaction was observed, thus lending more validity to the previous cocaine response in females treated with CP55,940.

A third set of animals consisting of female CP55,940-exposed (n = 5), male CP55,940-exposed (n = 6), female vehicle-exposed (n = 5) and male vehicle-exposed (n = 5) rats were analysed for glucose metabolism in the brain. These animals followed the same protocols as the previous groups. The difference in this experiment occurred on postnatal day 100 when animals were injected with 2-deoxy-2[^18^F]-fluoro-D-glucose (FDG) and subsequently analysed via positron emission tomography (PET). Specifically, PET further corroborated results found in the CP55,940-treated females, with a demonstrable hypoactivation of the amygdalo-enthorinal area with simultaneous hyperactivation of the frontal cortex observed. This activity was not observable in males. The results obtained in this instance point further to the differential effects of adolescent cannabinoid exposure mediated by sex. Previous studies have also observed such sex-specific effects when both genders were included in analyses. 

Research analysing the effects of cannabinoid exposure on subsequent cocaine activity has utilised metabolic approaches which allow for the development of more specific insights into the region-specific effect of cocaine. For instance, researchers have analysed the brains metabolic responses to cocaine in both male and female Wistar rats exposed to CP55,940 during adolescence which resulted in a region specific understanding of drug metabolism [132]. To achieve this, Wistar males and females were mated with subsequent litters gender balanced and utilised for experimentation. Pups were then weaned on postnatal day 22 and housed in groups dependent on sex. In these groups, the pups were administered CP55,940 (0.4 mg/kg) or vehicle (ethanol:cremaphor:saline [1:18:18]) once daily intraperitoneally from postnatal day 28–38. Four experimental groups were thus established: CP55,940-treated males, CP55,940-treated females, vehicle-treated males and vehicle-treated females. Animals were then housed individually from postnatal day 75 onwards and prepared for subsequent analyses. PET imaging was conducted in two different sessions. One session followed saline administration on postnatal day 100. The second session was conducted following amphetamine administration on postnatal day 102. After both administration procedures, FDG was injected into exposed rats. Tomographic images were subsequently constructed. Results revealed no differences in baseline metabolism amongst male Wistar pups exposed to CP55,940. After cocaine challenge, lower metabolism was revealed in the dorsal striatum of vehicle males, with these alterations not observable in CP55,940-treated males. In female Wistars, CP55,940 treatment was associated with higher baseline metabolism compared to their vehicle-treated counterparts. Interestingly, these differences were eliminated after cocaine challenge. Vehicle-treated females demonstrated no alterations in FDG uptake after cocaine administration. 

Exposure to the potent CB_1_ receptor agonist HU-210 has also been analysed for its perturbing effects on future drug activity. Researchers specifically sought to determine the effects of escalating HU-210 doses within adolescent male and female Sprague Dawley rats [46]. On postnatal day 35, male and female rats received intraperitoneal injections of escalating HU-210 doses for 12 consecutive days (Day 35–38: 25 µg/kg, Day 39–42: 50 µg/kg, Day 43–46: 100 µg/kg). Another experimental group received vehicle treatment to act as a control. Rats were subsequently left drug-free and undisturbed after this treatment period until postnatal day 70. Male and female rodents were then subjected to an acute-restraint stress challenge on postnatal day 75. After being restrained, blood samples were obtained and analysed. Results demonstrated significant increases in the hypothalamo-pituitary axis (HPA) response as well as increases in corticosterone levels within males but not females.

An amphetamine sensitisation paradigm was also conducted by the same researchers [46]. Rats from the acute-restraint test were utilised in this experiment with numerous experimental groups. All groups received either amphetamine or vehicle treatment from postnatal day 105–120. For the first 8 days of this period animals received 1 mg/kg administrations. For the remaining 8 days animals received 2 mg/kg administrations. After the administration schedule was completed, animals were left undisturbed for 2 weeks. After this rest period, they were administered one final 1 mg/kg dose of either amphetamine or vehicle on testing day. Rat behaviour was subsequently observed. Analyses revealed that HU-210 exposure increases amphetamine-related stereotypical behaviour in adult females, but not males. While significant differences were observed in stereotypic behaviour, no differences were observed across sexes in rearing behaviour. The results further point to the differential effects that cannabinoid exposure can seemingly have across sex. Females in this instance were more sensitive to amphetamines. 

## 3. Discussion

Included in this review were experiments which aimed to analyse the effects of developmental cannabinoid exposure on the subsequent behavioural and molecular activity of opioids, amphetamines and alcohol. While significant molecular and behavioural effects were observed across perinatal and adolescent periods, some observations provided counterevidence. It is clear that cannabinoid exposure affects development, however, it remains unclear exactly how this impact on development affects the future activity of other drugs. Studies included in this review demonstrate the equivocal nature of conclusions in this area.

Firstly, studies which focused on the perinatal period demonstrated varied behavioural and molecular effects that cannabinoid exposure during this period appeared to facilitate. Focusing on opiates, the evidence pointed to significant molecular and behavioural effects of Δ^9^-THC on subsequent opiate activities. Specifically, exposure to Δ^9^-THC appeared to affect the molecular activity of opiate receptors within male reward pathways with concomitant effects on the behaviour of these male rats toward opiate stimuli [38,39]. Other investigations highlighted that these molecular and behavioural effects were not observed in female rodents [94,118]. Illustrated here is the confounding effect of sex. Sex was observed consistently as a confounding factor in studies which acknowledged it as an experimental variable. This was due to myriad reasons, with recent research providing one broad explanation through analysis of the pivotal role that the estrogen system plays in concert with the ECS [133]. For example, the key estrogen system functional unit estradiol has been implicated in the expression of FAAH, which is a regulatory enzyme key to ECS function. With estrogen system components implicated in the expression of ECS components, a plausible mechanism for the sex-related differences observed in studies contained within this review becomes evident [133]. With the existence of clear sex differences, research should have delineated between sex, however, a large limitation of the studies included in this review was their disregard of sex-specific variance with a lack of sufficient exploration of sex-dependent responses when they were observed by the researchers.

More specific explanations for the observed sex differences in opiate studies appear to centre around dopaminergic output. Some investigators suggested that the lack of effect observed in females may correspond with naturally higher dopaminergic activity in the NAcc prior to the onset of any opiate administration. However, a recent investigation has pointed to a general sexual congruency within dopaminergic output, particularly in the NAcc of rats [134]. With this congruency established, it is more likely that sex related dopaminergic differences are mediated by mechanisms secondary to the dopaminergic system [134]. Investigations included in this review highlighted that female rodents may be more susceptible to opiates as a result of sex differences in dopaminergic activity. However, in light of recent conclusions, it would be pragmatic to replicate these studies and focus specifically on the secondary regulation of dopaminergic activity, rather than solely on the dopaminergic output of one particular brain region. Such a focus would provide a more up-to-date observation of the sex-specific effects that perinatal Δ^9^-THC exposure causes.

In terms of the behavioural effects observed in males which manifested across behavioural paradigms, researchers suggested varied molecular explanations for such observations. In certain instances, it appeared that Δ^9^-THC exposure significantly affected mRNA expression of opioid-related units during the prenatal period, with these alterations affecting the opioid system long into adulthood. This explanation received recent validation with other paradigms also demonstrating a proclivity of Δ^9^-THC to differentially alter the mRNA expression of opioid peptides and receptors [135]. Such molecular alterations may reflect the secondary means of dopaminergic activity regulation previously mentioned. This secondary regulation of dopaminergic activity is also hinted at when researchers observed increases and concomitant decreases in key developmental molecules such as RNA polymerase II. Fluctuation of these molecules observed specifically at the dopamine receptor 2 gene locus as a result of perinatal Δ^9^-THC exposure provides further evidence of potential secondary regulation of dopaminergic output. The epigenome has been shown to mediate various developmental perturbations [136], and may be mediating exposures to Δ^9^-THC in a secondary manner during the perinatal period, ultimately affecting dopaminergic output. 

A key limitation of research included in this review was the lack of studies specifically targeting the previously mentioned secondary regulators. The importance of this secondary regulation can be highlighted by a brief focus on research analysing epigenetic modifications of the ECS. Processes such as DNA methylation and histone acetylation/deacetylation have been implicated in reproductive processes with knock-on effects for disease pathogenesis and progression [137]. The ECS itself has been implicated in mediating these aforementioned processes, specifically within studies focusing on drugs of abuse. For example, it has been demonstrated that external cues such as alcohol consumption can induce DNA methylation in mice, resulting in the genotypes of subsequent offspring being more susceptible to alcohol-related disorders [138]. Interestingly, the previous research highlighted the key role of the ECS in mediating such epigenetic responses to alcohol with CB_1_ knock-out mice protected against alcohol-induced impairments [138]. Evident here is the role of the epigenome in mediating external cues and internal responses, with the key role of the ECS in mediating these processes. Going forward, there should be more focus on the specific epigenetic responses to various drugs of abuse, including native ECS modulators such as Δ^9^-THC. With the ECS implicated in regulating epigenetic responses themselves, exogenous insults which dysregulate the ECS should be the topic of further research. 

Collated investigations into opiate-related activity included in this review are subject to caveats. The main consideration is the varied nature of Δ^9^-THC dosage utilised. Investigations included in this review used varied dosages (ranging from 0.15 mg/kg–5.0 mg/kg). Observations across sex would have increased validity if a specific dosage was adhered to consistently. These large dose ranges are a significant limitation on our ability to be consistent in conclusions. Any future investigations in the area should aim to utilise similar dosages as well as administration routes. A relative strength of the included opiate-related studies investigating the perinatal period is that they all utilised Δ^9^-THC. This allows for consistency in conclusions and increases the translatability of results to humans, where Δ^9^-THC is the most commonly used cannabinoid compound.

The effects of perinatal cannabinoid exposure on subsequent amphetamine and cocaine responses were also analysed. Significant effects of perinatal Δ^9^-THC exposure were observed on subsequent amphetamine responses. It was observed that Δ^9^-THC-exposed pups had a dampened locomotor response to amphetamines across sexes [43]. Researchers suggested that the activation of CB_1_ receptors dampened increases in dopaminergic output within key reward pathways [139,140,141]. It is important to highlight that this result stands alone with no corroborating results from the perinatal period present in this review. One study found no effect of the FAAH inhibitor URB597 on subsequent cocaine-related activity in males [44]. However, while providing contrasting evidence in terms of overall effect on amphetamine response, the mechanism of action by which URB597 acts is dissimilar to the hypothesised mechanism through which Δ^9^-THC exerts its effects [142,143]. Therefore, to utilise this observation toward negating the effects of Δ^9^-THC is tenuous. This highlights a relevant limitation of studies on amphetamines, namely their use of varied cannabinoid compounds in experimental design. In contrast to opiate-related studies where the consistent use of Δ^9^-THC was a strength, amphetamine studies were characterised by inconsistencies in the cannabinoid being used. This was a major limitation and severely reduced generalisability of results. What was also highlighted was the dearth of specific investigations analysing the effects of perinatal cannabinoid (particularly CB_1_ agonists) exposure on the subsequent amphetamine response. While results with Δ^9^-THC and URB597 point to potential effects on subsequent responses to amphetamines, more investigation in this area is warranted, especially with the predicted increases in societal cannabis use considered [144,145].

The effects of perinatal cannabinoid exposure on subsequent alcohol-related behaviours have also been analysed, revealing no statistically significant effects of exposure on subsequent rat behaviour toward alcohol stimuli [42]. While no statistically significant behavioural effects of perinatal Δ^9^-THC exposure were observed via SA paradigms, molecular analyses did reveal 112 genes differentially altered by the Δ^9^-THC treatments. Again, the alteration of genes as a result of Δ^9^-THC exposure points to underlying molecular effects which may manifest in behavioural paradigms over longer periods of time [146]. Most studies included in this review took an acute perspective which is a limitation that must be addressed. This points to the necessity of further investigation into the area with experiments potentially considering longer time frames. If sex-specific effects are also considered, these results relating to the effects of Δ^9^-THC on future alcohol SA were only for males. Further investigation across genders may reveal important insights. 

Investigations which focused on the adolescent period demonstrated varied effects of cannabinoid exposure on the subsequent activity of opioids and amphetamines as measured by behavioural and molecular assays. A characteristic of research investigating adolescent exposure is the broad range of cannabinoid molecules utilised within experimental designs. In contrast to perinatal investigations which predominantly utilised Δ^9^-THC, studies of adolescents used Δ^9^-THC, as well as WIN5521-2, CP55,940 and HU210. While it is important to use various molecules, consistent conclusions do not exist for any one of them as of yet. This is a critical limitation to our understanding of the adolescent period. Future research should aim to consistently apply the same molecule to experimental designs, thus building a consistent and useful profile over time.

Δ^9^-THC has shown mixed results regarding its effects on subsequent opiate activity at the behavioural and molecular level. Across paradigms, Δ^9^-THC exposure in adolescence was observed to affect subsequent heroin seeking and the rewarding effects of heroin in male Wistar rats and male Lewis rats [41,124]. This directly contrasts with results obtained in perinatal investigations. In male Long–Evans rats, adolescent Δ^9^-THC exposure was shown to have a significant effect on subsequent opiate intake and the activity of opioid neuron populations in the limbic system [40]. This result was echoed within Fischer 344 males [124], thus supporting results obtained in the perinatal period. Researchers postulated that Δ^9^-THC potentially affected opioid receptor activity in key reward pathways. This explanation received further validation from recent research, pointing to the efficacy of Δ^9^-THC as a negative allosteric modulator of opioid receptors [147]. It is worth noting that investigations into the female adolescent response were sparse in the literature, with the sex-specific effects receiving a minor focus across the adolescent period. Overall, the effects of Δ^9^-THC exposure in adolescence on subsequent opioid intake, seeking and reward remain equivocal. These equivocal results were observed across a broad range of dosages (1.5–10 mg/kg), but were limited to one sex (male). Focus on one sex again is a limitation. Any investigations planning on analysing these phenomena further should ensure the inclusion of both sexes, or alternatively conduct future investigations solely on female rodents. The inclusion of both sexes should be common practice due to the well-established confounding effects of sex.

Adolescent exposure to other cannabinoid agonists was also analysed. For example, the cannabinoid agonist CP55,940 was observed to alter opioid SA in a sex-dependent manner [125]. Wistar males exposed to CP55,940 throughout adolescence demonstrated significantly higher SA patterns. Conversely, in females, exposure did not modify opioid SA. CP55,940 exposure did appear to directly affect the endogenous opioid system, specifically opioid receptor activity in the NAcc. Effects of synthetic cannabinoid agonists have been recently translated into human populations, where use of synthetic cannabinoids prolonged withdrawal from opioids [148]. This investigation highlights that observations pertaining to synthetic cannabinoids and opioid receptors may transcend animal models. 

Adolescent exposure to WIN55,212-2 was also analysed through a transgenerational lens [126,127]. Observations pointed to the fact that adolescent offspring exposed to WIN55,212-2 by transgenerational means subsequently exhibit high sensitivity to opiates in the future. These results were replicated and extended to include female rodents, with WIN55,212-2 exposed offspring demonstrating higher levels of opioid receptors when compared to vehicle treated offspring. Synthetic cannabinoids encountered in utero also appear to act as effectors of the epigenome, resulting in an array of molecular alterations which ultimately shape how an animal may phenotypically react to a specific drug [136]. Again, in this instance, the epigenome appears to be regulating subsequent drug activity in a secondary manner. The transgenerational effects of adolescent WIN55,212-2 exposure observed here further implicate epigenetic mechanisms in the perturbing effects of cannabinoids. 

The amphetamine response has also been the subject of investigation with WIN55,212-2 analysed to determine its effects on the future activity of amphetamines. Rodents exposed to WIN55,212-2 during adolescence presented with significantly less inhibition after amphetamine administration [123]. These results demonstrated a developed tolerance to amphetamines as a result of exposure. A recent study has demonstrated that cannabinoid mechanisms play a significant role in reinstating amphetamine-related behaviours [149]. The previously mentioned result echoes this. While significant effects have been found, other observations [128] point to no significant effects of WIN55,212-2 exposure on subsequent locomotor activity and stereotypical behaviour. In such investigations, WIN55,212-2 exposure appeared to cause a tendency towards reduced locomotor activity when challenged with amphetamine and cocaine, however, these observations were not deemed statistically significant [128,129]. The variation in results obtained may be explained by the dosages of WIN55,212-2 utilised. Experiments which achieved statistically significant effects utilised higher dose ranges of WIN55,212-2 (2–8 mg/kg) when compared to experiments which did not achieve significant effects (0.625–2.5 mg/kg). However, recent research in adult rodents utilised lower dosages of WIN55,212-2 (0.2–0.8 mg/kg) and still achieved significant effects on the outcome measures being analysed [150]. Therefore, it is unclear whether dosage in this instance is the limiting factor.

Adolescent Δ^9^-THC exposure was also analysed in order to determine its ability to affect subsequent amphetamine responses. No effects of Δ^9^-THC on subsequent amphetamine locomotor responses were observed [128], however, Δ^9^-THC was shown to reduce the rewarding effects of amphetamines at differing dosages [130]. These mixed effects observed within amphetamine-related outcomes are interesting and it is important to note that these studies were solely carried out on males. While delineating consistent responses in males is important, sex-specific responses should also be incorporated. The sole focus on males in this instance is a limitation [134]. Due to predicted future increases in female cannabis usage, more studies should be carried out in females in order to address this gap. Interestingly, the effect of adolescent Δ^9^-THC on the rewarding effect of Δ^9^-THC in the future has also been analysed [130], with observations demonstrating that a lower dose had a significant effect (0.1 mg/kg) while a larger dose of Δ^9^-THC did not (1.0 mg/kg). Future research should begin to focus on the future effects of other commonly used drugs, specifically drugs currently underrepresented in the literature such as Δ^9^-THC.

Other cannabinoids were also analysed in an effort to determine their ability to affect future amphetamine responses. Research on CP55,940 and HU-210 generated unique data across genders where metabolic responses to amphetamine insults were analysed. CP55,940 was observed to differentially affect cocaine metabolism of females in comparison to males [131,132]. Females in CP55,940-treated groups were observed to self-administer a significantly greater amount of cocaine, with this sensitivity in females further replicated when rodents were exposed to HU-210 [46]. These observed sex differences were explained in various ways, however, recent research specifically examining differential sex effects concluded that the most plausible explanation was a variation in time intervals during which cannabinoids were administered [151]. Here, the differential effect of sex is highlighted further.

## 4. Conclusions

In conclusion, experiments have been presented investigating the effects that developmental cannabinoid exposure can have on the subsequent activity of other drugs within preclinical rodent populations. While significant effects have been observed at various junctures, it would appear that significant counterevidence also exists. Therefore, a comprehensive conclusion regarding the consistent effects that developmental cannabinoid exposure may have on future drug activity cannot be reached. A sparsity of research specifically investigating the phenomena within key developmental windows exists and needs to be addressed. Recent research has analysed the area, however, a focus on adults steers the critical lens away from important developmental windows [150]. Following on from developmental windows, another area of growing importance is the transgenerational effects cannabinoids may impose. In the research that has been conducted, inconsistencies in the management of and/or lack of representation regarding the inevitable sex effects further add to our inability to be conclusive. Studies incorporating sex still remain a minority; with the inclusion of both sexes enriching our understanding, it is imperative to do so going forward [151,152]. Additionally, the varied utilisation of differing cannabinoid agonists in experiments contained in this review takes away from the overall translatability of results. Cannabis is made up of hundreds of phytocannabinoid compounds which are all in dynamic relationships with one another, thus affecting each other’s activity when ingested [153]. Through solely utilising Δ^9^-THC and its synthetic analogues, research may not be capturing the true effects of developmental cannabis exposure [136]. In future experiments, the dynamic relationship of phytocannabinoid compounds should also be captured to further improve the clinical translatability of results. 

## Figures and Tables

**Table 1:** Brief description of behavioural paradigms.

Behavioural Paradigm	Description	Reference
Self-Administration (SA)	This paradigm is based on operant conditioning. Animals are taught through practice, and subsequently learn to conduct certain actions that result in self-administering a reinforcer (drug of abuse). Then, reinforcement is the event that follows the response (e.g., lever press) and increases the probability of responses reoccuring.	[110]
Conditioned Place Preference (CPP)	This paradigm results in a specific environment being paired with drug administration and vehicle administration seperately. After such pairing occurs, animals are placed back in the environment in a drug free state and their preference for each environment is tested.	[110]
Elevated Plus Maze (EPM)	Rodents are placed at the junction of a four-arm maze and their entry into the particular arms is measured by recording apparatus. Time spent (duration and entries) in open arms reflects anti-anxiety behaviour.	[111]
Intracranial Self-Stimulation (ICSS)	Animals learn to deliver elctrical pulses into specific regions of their own brains which are implicated in reward processes. The acute administration of various drugs of abuse including cocaine, amphetamine and opiates lowers ICSS thresholds in experimental animals, indicating a reward-facilitating effect. Withdrawal from administration of these compounds creates increases in ICSS thresholds which results in the manifestation of a withdrawal state. Through the dynamic orchestration of these states, researchers can understand reward-related behaviours further.	[112]

**Table 2:** Effects of perinatal cannabinoid exposure on subsequent drug use.

Cannabinoid	Dose	Strains	Gender	Effect	Drug of Abuse	Reference
Δ^9^-THC	5 mg/kg	Wistars rats	F	✗	Morphine	[94]
Δ^9^-THC	0.15 mg/kg	Long–Evans rats	M	✓	Heroin	[39]
Δ^9^-THC	0.15 mg/kg	Long–Evans rats	M	✓	Morphine	[38]
Δ^9^-THC	1.5 mg/kg	Long–Evans rats	M&F	M✗ F✗	Heroin	[118]
Δ^9^-THC	0.15 mg/kg	Sprague Dawley rats	M&F	M✓ F✓	Amphetamine	[43]
URB597	1, 3, 10 mg/kg	ICR mice	M	✗	Cocaine	[44]
Δ^9^-THC	5.0 mg/kg	Wistar rats	M	✗	Ethanol	[42]
Δ^9^-THC	5.0 mg/kg	Wistar rats	M	✗	Ethanol	[42]

(✗): no significant effect of cannabinoids on effect(s) of another drug. (✓): significant effect of cannabinoids on the effect(s) of another drug. (M): male. (F): female. (ICR) Institute of Cancer Research.

**Table 3:** Equivalence of rat gestational period to mouse gestational period and human gestational period.

Rat (G21.5)	Mouse (G18.5)	Human (Limbic) (G270)	Human (Cortex) (G270)
Day 9.0	Day 8.3	Day 23	Day 29
Day 9.5	Day 8.7	Day 26	Day 32
Day 10.0	Day 9.1	Day 28	Day 35
Day 10.5	Day 9.5	Day 30	Day 39
Day 11.0	Day 9.9	Day 33	Day 42
Day 11.5	Day 10.3	Day 35	Day 45
Day 12.0	Day 10.7	Day 38	Day 48
Day 12.5	Day 11.1	Day 40	Day 52
Day 13.0	Day 11.5	Day 43	Day 55
Day 13.5	Day 11.9	Day 45	Day 58
Day 14.0	Day 12.3	Day 47	Day 61
Day 14.5	Day 12.7	Day 50	Day 65
Day 15.0	Day 13.2	Day 52	Day 68
Day 15.5	Day 13.6	Day 55	Day 71
Day 16.0	Day 14.0	Day 57	Day 74
Day 16.5	Day 14.4	Day 60	Day 78
Day 17.0	Day 14.8	Day 62	Day 81
Day 17.5	Day 15.2	Day 64	Day 84
Day 18.0	Day 15.6	Day 67	Day 87
Day 18.5	Day 16.0	Day 69	Day 91
Day 19.0	Day 16.4	Day 72	Day 94
Day 19.5	Day 16.8	Day 74	Day 97
Day 20.0	Day 17.2	Day 77	Day 100
Day 20.5	Day 17.6	Day 79	Day 104
Day 21.0	Day 18.0	Day 82	Day 107
Birth	Birth	Day 84	Day 110

This table is adapted with permission from Clancy et al. (2001) (License #: 5146960407020; License date: 13 September 2021) and presents the rat gestational period (Column 1) with the equivalent gestational timetables of the mouse and humans in relation to the rat [120].

**Table 4:** Effects of adolescent cannabinoid exposure on subsequent drug use.

Cannabinoid	Dose	Strains	Gender	Effect	Drug of Abuse	Reference
WIN55,212-2	2, 4, 8 mg/kg	Sprague Dawley rats	M and F	M✓ F✓	Morphine/Amphetamine	[123]
Δ^9^-THC	1.5 mg/kg	Long–Evans rats	M	✓	Heroin	[40]
CP-55,940	0.2, 0.4 mg/kg	Wistars rats	M and F	M✓ F✗	Morphine	[125]
WIN55,212-2	1, 2, 4 mg/kg	Sprague Dawley rats	M	✓	Morphine	[126]
WIN55,212-2	1, 2, 4 mg/kg	Sprague Dawley rats	F	✓	Morphine	[127]
Δ^9^-THC	2.5, 5.0, 10 mg/kg	Wistar rats	M	✗	Heroin	[41]
Δ^9^-THC	2.0, 4.0, 8.0 mg/kg	Fischer 344 and Lewis rats	M	Fischer 344✓ Lewis rats✗	Heroin	[124]
WIN55,212-2	1.25 mg/kg	Sprague Dawley	M	✗	Amphetamine	[128]
WIN55,212-2	0.0625, 1.25, 2.5 mg/kg	Sprague Dawley	M	✗	Amphetamine	[128]
WIN55,212-2	2, 4, 8 mg/kg	Sprague Dawley	M	✗	Cocaine	[129]
Δ^9^-THC	0.075, 1.5, 3.0 mg/kg	Sprague Dawley	M	✗	Amphetamine	[128]
Δ^9^-THC	1.5 mg/kg	Sprague Dawley	M	✗	Amphetamine	[128]
Δ^9^-THC	0.1 and 1.0 mg/kg	Sprague Dawley	M	✓	Amphetamine	[130]
Δ^9^-THC	0.1 and 1.0 mg/kg	Sprague Dawley	M	✓	Δ^9^-THC	[130]
CP-55,940	0.4 mg/kg	Wistar rats	M and F	M✗ F✓	Cocaine	[131]
CP-55,940	0.4 mg/kg	Wistar rats	M and F	M✗ F✓	Cocaine	[131]
CP-55,940	0.4 mg/kg	Wistar rats	M and F	M✗ F✓	Cocaine	[132]
HU-210	25, 50, 100 µg/kg	Sprague Dawley rats (Exp 1)	M and F	M✓ F✗	Amphetamine	[46]
HU-210	25, 50, 100 µg/kg	Sprague Dawley rats (Exp 2)	M and F	M✗ F✓	Amphetamine	[46]

(✗): no significant effect of cannabinoid on activity of another drug. (✓): significant effect of cannabinoid on activity of another drug. (M): male. (F): female.

## Data Availability

Not applicable.
